# Significance of the Balance between Regulatory T (Treg) and T Helper 17 (Th17) Cells during Hepatitis B Virus Related Liver Fibrosis

**DOI:** 10.1371/journal.pone.0039307

**Published:** 2012-06-20

**Authors:** Jing Li, Shuang-Jian Qiu, Wei-Min She, Fu-Ping Wang, Hong Gao, Lei Li, Chuan-Tao Tu, Ji-Yao Wang, Xi-Zhong Shen, Wei Jiang

**Affiliations:** 1 Department of Gastroenterology, Zhongshan Hospital, Fudan University, Shanghai, China; 2 Liver Cancer Institute, Zhongshan Hospital, Fudan University, Shanghai, China; National Institute of Infectious Diseases, Japan

## Abstract

**Background:**

Hepatitis B virus-related liver fibrosis (HBV-LF) always progresses from inflammation to fibrosis. However, the relationship between these two pathological conditions is not fully understood. Here, it is postulated that the balance between regulatory T (Treg) cells and T helper 17 (Th17) cells as an indicator of inflammation may predict fibrosis progression of HBV-LF.

**Methodology/Principal Findings:**

The frequencies and phenotypes of peripheral Treg and Th17 cells of seventy-seven HBeAg-positive chronic hepatitis B (CHB) patients who underwent liver biopsies and thirty healthy controls were determined by flow cytometry. In the periphery of CHB patients, both Treg and Th17 frequencies were significantly increased and correlated, and a lower Treg/Th17 ratio always indicated more liver injury and fibrosis progression. To investigate exact effects of Treg and Th17 cells during HBV-LF, a series of *in vitro* experiments were performed using purified CD4^+^, CD4^+^CD25^+^, or CD4^+^CD25^−^ cells from the periphery, primary human hepatic stellate cells (HSCs) isolated from healthy liver specimens, human recombinant interleukin (IL)-17 cytokine, anti-IL-17 antibody and HBcAg. In response to HBcAg, CD4^+^CD25^+^ cells significantly inhibited cell proliferation and cytokine production (especially IL-17 and IL-22) by CD4^+^CD25^−^ cells in cell-contact and dose-dependent manners. In addition, CD4^+^ cells from CHB patients, compared to those from HC subjects, dramatically promoted proliferation and activation of human HSCs. Moreover, in a dramatically dose-dependent manner, CD4^+^CD25^+^ cells from CHB patients inhibited, whereas recombinant IL-17 response promoted the proliferation and activation of HSCs. Finally, *in vivo* evidence about effects of Treg/Th17 balance during liver fibrosis was obtained in concanavalin A-induced mouse fibrosis models via depletion of CD25^+^ or IL-17^+^ cells, and it’s observed that CD25 depletion promoted, whereas IL-17 depletion, alleviated liver injury and fibrosis progression.

**Conclusions/Significance:**

The Treg/Th17 balance might influence fibrosis progression in HBV-LF via increase of liver injury and promotion of HSCs activation.

## Introduction

Worldwide, hepatitis B virus (HBV) affects over 350 million individuals and continues to cause more than a million deaths annually from end-stage liver diseases [1].Although HBV itself is noncytopathic, it causes chronic immune-induced liver injury and forces disease progression from mild inflammation, to severe inflammation, to fibrosis, and finally to cirrhosis. Despite the close association of inflammation with fibrosis in HBV-related liver fibrosis (HBV-LF), little is known about cellular cross-talks between these two pathways.

Many mechanisms have been proposed for impaired virus-specific T cell responses during chronic HBV infection. One possible mechanism is induction of host-mediated regulatory mechanisms after exposure to HBV-related antigens. The most recent concerns regulatory T (Treg) cells, a subset of CD4^+^ cells suppressing immune responses to maintain unresponsiveness to self-antigens and prevent excessive immune responses to foreign antigens, which play an important role in autoimmune and infectious diseases [2]. These cells can be generated in the thymus as naturally-occurred Treg or in the periphery as induced Treg. Different populations of Treg cells have also been reported on the basis of high expression of CD25 and forkhead family transcription factor 3 (Foxp3) or on the basis of the production of immunosuppressive cytokines, such as interleukin (IL)-10 or transforming growth factor (TGF)-β [2]. CD4^+^CD25^+^Foxp3^+^ cells are the most characterized Treg cells. Although these Treg cells are also characteristic of the expression of cytotoxic T-lymphocyte antigen 4 (CTLA-4)/CD152, CD45RO and glucocorticoid-induced tumor necrosis factor-related protein (GITR), Foxp3 has been demonstrated to be a unique marker. In humans, CD4^+^CD25^+^Foxp3^+^ cells represent 3–10% of total CD4^+^ cells in peripheral blood [3]. CD4^+^CD25^+^Foxp3^+^ cells have recently been reported to increase in chronic hepatitis B (CHB) patients, which could inhibit HBV-specific CD8^+^ T cell response and show a close association with HBV loads and serum alanine aminotransferase (ALT) levels [4]–[6]. Here, we suppose Treg cells to be a ‘dual-edged’ sword during chronic HBV infection for being detrimental to facilitate HBV escape and being protective to prevent immune-mediated liver injury.

Recent researches on Treg cells have turned attention to their interactions with other effector cells because their balance determines the outcome of immune and inflammation. Interestingly, T helper 17 (Th17) cells, another newly identified subset of CD4^+^ cells with retinoid orphan nuclear receptor γ t (RORγt) as the specific transcriptional factor, are closely-linked with Treg cells and have also been implicated in autoimmune and infectious diseases [7]. On development pathways, both induced Treg and Th17 cells require TGF-β. Moreover, retinoic acid (RA) and IL-2 promote the development of Treg cells and inhibit that of Th17 cells, whereas IL-6, IL-21 and IL-23 facilitate the development of Th17 cells and inhibit that of Treg cells [8]. On immune function, in contrast to the regulatory features of Treg cells, Th17 cells mediate strong inflammation by producing a cocktail of cytokines such as IL-17, IL-17F, IL-21, IL-22, IL-6, and tumor necrosis factor-α (TNF-α), of which IL-17 has been characterized as a major effector cytokine [7]. Increase of Th17 cells have also been observed in CHB patients to inhibit HBV replication and cause immune-mediated liver injury [9]–[10]. Accumulating data suggest the plasticity of Treg and Th17 cells under certain cytokine conditions. For example, Treg cells can be converted to IL-17-expressing cells in response to IL-6 and IL-21 whereas Th17 cells can give a rise to Th1 or Th2 cells in the presence of IL-12 or IL-4, respectively [11]. Thus, we suggest the balance (or interplay) between Treg and Th17 cells might be a crucial indicator of immune homeostasis that can partly reflect the balance between pro-inflammation and anti-inflammation. The Treg/Th17 balance has already been reported to skew in several autoimmune and inflammatory diseases such as primary biliary cirrhosis and rheumatoid arthritis [12], [13]. Also, Treg/Th17 balance has been described to change in CHB patients undergoing treatment with entecavir [10] or end-stage liver failure [14]. However, whether and how this balance influences disease progression of HBV-LF has not been determined.

Hepatic stellate cells (HSCs), located in the space of Disse, are the cornerstone of liver fibrosis [15]. In response to inflammatory stimuli, quiescent HSCs usually activate and transform into myfibroblast-like cells and produce abundant cytokines and extracellular matrix (ECM). Besides, activated HSCs secret matrix metalloproteinases (MMPs) to degrade ECM and also synthesize tissue inhibitors of metalloproteinases (TIMPs) to control the enzyme activity of MMPs. Thus, to some extent, the functional status of HSCs determines whether liver fibrosis progresses or regresses. Collectively, to estimate whether a factor is protective or detrimental during liver fibrosis, researches could be started by getting a preliminary picture from *in vitro* co-culture/stimulation experiments with HSCs. Interestingly, HSCs are no longer bystanders of inflammation, and are recently reported to have various immune functions. Under inflammatory circumstances, activated HSCs can behave as antigen presenting cells and elicit T cell proliferation [16], [17], which requires direct contacts between T cells and HSCs. Moreover, additional expression of programmed cell death ligand 1 (PD-L1) endows HSCs with the immune regulatory property to induce T cell apoptosis [18], [19]. This dual roles of HSCs as both fibrotic and inflammatory participants make their interplay with other immune cells as a perfect cut-in point to investigate potential cellular cross-talks between inflammation and fibrosis [20]. HBV-LF is essentially an immune and inflammatory process. However, whether and how immune cells such as Treg and Th17 cells influence fibrosis progression is unclear.

Here, we designed this study to determine the Treg/Th17 balance in CHB patients, and to ascertain whether this balance influences fibrosis progression of HBV-LF on their influence on HBV replication, immune-mediated liver injury, and HSCs activation.

## Methods

### Patients

Seventy-seven CHB patients who underwent liver biopsies at Zhongshan Hospital (Shanghai, China) and thirty healthy controls (HC) were enrolled. All patients were seropositive for hepatitis B surface antigen (HBsAg) and hepatitis B e antigen (HBeAg), and were diagnosed according to criteria previously described [21]. Patients with other viral infections, fatty liver diseases or autoimmune liver diseases were excluded. None of these patients had received anti-viral or immunomodulatory therapy before sampling. The serum HBV loads were detected by a virological assay (detection limit: 500 copies/mL) previously described [10]. The inflammation grades (G stands for inflammation) and fibrosis stages (S stands for fibrosis) of liver biopsies were determined by two senior clinical pathologists using a modified Scheuer scoring system [22]. The study protocol was approved by the Research Ethics Committee of Zhongshan Hospital (No. 2010-87) and written informed consent was obtained from each subject. The clinical characteristics of all subjects are listed in Table 1. To investigate how Treg and Th17 cells involve in CHB pathogenesis, CHB patients were further divided into several subgroups according to their levels of liver injury or HBV replication, or fibrosis progression to be analyzed. First, patients with the serum HBV load of 10^5^–10^7^ copies/mL (*n* = 57) were divided into three subgroups according to the serum ALT level: < upper limit of normal (ULN) (*n* = 7), ULN–2ULN (*n* = 15) and >2ULN (*n* = 35). Second, patients with the normal ALT (*n* = 13) were divided into two subgroups according to the HBV load: 10^5^–10^7^ copies/mL (*n* = 7) and >10^7^ copies/mL (*n* = 6). Third, patients with the inflammation grade of G2–3 (*n* = 56) were divided into the following two subgroups according to the fibrosis stage (S score): S0–2 (*n* = 21) and S3–4 (*n* = 35). Also, to dissect the association of the Treg/Th17 ratio with degree of hepatic inflammation, CHB patients with fibrosis stages of S0–1 (n = 15) were divided into two subgroups according to their inflammation grades (G score): G1 (n = 6) and G2–3 (n = 9).

**Table 1:** Clinical characteristics of all subjects.

	Healthy controls	Chronic hepatitis B
**Case**	30	77
**Age (years)**	32 (25–47)	39 (30–49)
**Sex (M/F)**	23/7	60/17
**AST (U/L)**	45 (30–60)	152 (62–320)
**ALT (U/L)**	39 (24–58)	198 (55–560)
**TBIL (µmol/L)**	10 (4–18)	19 (13–32)
**PT (s)**	NA	12 (10–13)
**ALB (g/L)**	48(40–54)	40 (35–45)
**HBV DNA (copies/mL)**	NA	2×10^6^ (1×10^5^−6×10^8^)

Data are shown as median and range. AST, aspartate aminotransferase; ALT, alanine aminotransferase; TBIL, total bilirubin; PT, prothrombin time; ALB, albumin; NA, not applicable.

### Mouse Liver Fibrosis Models

Eight-week-old female BALB/c mice weighing around 18 g were obtained from the Shanghai Laboratory Animal Centre (Chinese Academy of Science, Shanghai, China), maintained under specific pathogen-free conditions and used for concanavalin A (ConA) induced fibrosis models. Liver fibrosis (ten mice in each group) was induced by intravenous (i.v.) injection with ConA (Sigma-Aldrich, Tokyo, Japan) dissolved in pyrogen-free phosphate buffered saline (PBS) at a dose of 10 mg/kg body weight once a week for up to 4 weeks [23], [24]. Control mice were injected with the same volume of PBS. Heparinized plasma samples and liver specimens were collected at week 1, 2, 3 and 4 after ConA administration for analyses. Liver injury was determined by measuring serum ALT levels using a commercially available ALT Reagent Kit (Rongsheng Biotech, Shanghai, China). For histopathologic analyses, 4 µm-thick sections of paraffin-embedded and formalin-fixed liver specimens were stained with Masson to determine the collagen fibers. Each assay was performed in triplicate. The experimental procedures conformed to the guidelines outlined in the Guide for the Care and Use of Laboratory Animals and were approved by the Research Ethics Committee of Zhongshan Hospital (No. 2010-87).

### Preparation of Peripheral Blood Mononuclear Cells

Peripheral blood mononuclear cells were isolated from heparinized blood samples using Ficoll-Paque PLUS (GE Healthcare, Piscataway, NJ, USA). Cells were cultured in RPMI 1640 medium (Gibco, Grand Island, NY, USA) containing 10% fetal calf serum (FCS, Gibco). IL-2 (300 U/mL, PeproTech, Rocky Hill, NJ, USA) was added to enable survival of the lymphocytes *in vitro*.

### Preparation of Non-parenchymal Cells from Mouse Livers and Spleens

For the preparation of non-parenchymal liver cells, the abdominal cavities of anesthetized mice were opened and the livers were perfused through the portal vein for 5 min with Hank’s balanced salt solution (HBSS), 4 min with 0.5 mg/mL pronase solution, and 4 min with 0.25 mg/mL collagenase IV solution at a flow rate of 6 mL/min [17]. The liver tissue was then minced and further digested in 50 mL HBSS supplemented with 50 mg collagenase IV, 50 mg pronase, and 1 mg DNase (20 min, 37°C). The resulting cell suspension was passed through a 100 µm nylon mesh filter and then centrifuged at 300 *g* (10 min, 4°C). The cell pellets were resuspended in 8 mL 40% Percoll solution and then carefully overlaid on 5 mL of 70% Percoll solution (Sigma-Aldrich, St. Louis, MO, USA). After centrifugation at 450 *g* (17 min, 4°C), the layer of cells at the intermediate interface was harvested as target cells. Spleen cells were isolated following mechanical disruption of the spleen and erythrocyte lysis as described elsewhere [25].

### Purification of Human CD4^+^, CD4^+^CD25^+^ and CD4^+^CD25^−^ Cells

When sufficient PBMCs could be obtained from G2–3S3–4 CHB patients, they were chosen for further purification. CD4^+^, CD4^+^CD25^+^ and CD4^+^CD25^−^ cells were purified using a fluorescence activated cell sorting (FACS) Aria cell sorter (Becton Dickinson, Palo Alto, CA, USA) after the staining with anti-CD4-fluorescein isothiocyanate (FITC) and/or anti-CD25-phycoerythrin (PE) antibodies. The purity of isolated cells was >90% (shown in Figure S1). Blood samples were obtained from CHB patients and their age-and-sex matched HC subjects. Co-cultures were performed with human leukocyte antigen (HLA)-compatible (HLA-identical, cells from the same individual) or incompatible (Allogenic, cells from the same species but different individuals) cells.

### Preparation of Human Hepatic Stellate Cells

Human HSCs were obtained from liver specimens of patients with liver hemangiomas who had undergone surgical resections. This material was used in accordance with the guidelines of the local Ethics Committee. HSCs were isolated using methods previously described [16]. They were cultured at a concentration of 1×10^5^ cells per well in high glucose Dulbecco’s modified Eagle’s medium (DMEM, Gibco) containing 20% FCS for 1–2 (quiescent), 3–5 (intermediate), or 7–10 (activated) days as described elsewhere [18], [26]. Cell viability was greater than 90% as determined by trypan blue exclusion. The purity of HSCs ranged from 90% to 95% as determined by glial fibrillary acidic protein (GFAP) staining (shown in Figure S2) and the typical microscopic appearance of the lipid droplets. On days 1–2, isolated HSCs were quiescent, round, had abundant lipid droplets, and lacked α-smooth muscle actin (α-SMA) expression. At day 7, the cells become activated and begin to express a-SMA (shown in Figure S2). Cells from days 3–5, which have an intermediate appearance, were chosen for *in vitro* analyses in this study.

### T-cell Proliferation Assay

Purified CD4^+^CD25^−^ cells were cultured in triplicate in a concentration of 1×10^5^ cells per well in 100 µL RPMI 1640 medium containing 10% FCS. The cells were stimulated with or without 1 µg/mL HBcAg (ProSpec, East Brunswick, NJ, USA) and cultured for 6 days. Inhibition of CD4^+^CD25^+^ cells on cell proliferation and cytokine production of CD4^+^CD25^−^ cells was tested by directly adding 10% (11,000 cells), 20% (25,000 cells), or 30% (43,000 cells) CD4^+^CD25^+^ cells to 1×10^5^ CD4^+^CD25^−^ cells as described elsewhere [4]. After 5 days of incubation, 50 µL of supernatant was harvested for analysis. Cell proliferation was measured by the thymidine method, and converted to a stimulation index as the mean number of counts per minute (cpm) for cells exposed to antigen divided by the mean number of cpm for cells not exposed to antigen.

### Cell Co-cultures

Using a HTS transwell-96 co-culture system (Corning, Corning, NY, USA), in which a polycarbonate membrane with a pore-size of 0.4 µm is set between upper and lower compartments to prevent direct cell contacts. Cells of different types can be adding to the same compartment or different compartments for contact or non-contact co-cultures. 1 µg/mL HBcAg was always added in each well during the co-cultures to provide specific antigen stimulation during HBV-LF *in* *vitro*.

### Detection of Cytokines

T-cell cytokines (IL-10, TGF-β, IL-17, IL-21, IL-22 and TNF-α) in serum samples, or in the supernatant of *in vitro* cultures were measured using enzyme-linked immunosorbent assay (ELISA) kits (R&D Systems, Minneapolis, MN, USA) according to the manufacturer’s instructions. The concentrations of cytokines (TGF-β, platelet derived growth factor [PDGF]-BB, connective tissue growth factor [CTGF] and epidermal growth factor [EGF]) principally produced by activated HSCs in the supernatant of *in* *vitro* cultures were also evaluated by ELISA kits (R&D Systems).

### Hepatic Stellate Cells Proliferation Assay

HSCs were directly co-cultured with human CD4^+^, CD4^+^CD25^+^ and CD4^+^CD25^−^ cells from age-and-sex matched HC and CHB subjects or incubated with human recombinant cytokine IL-17 (PeproTech) at various concentrations (1, 3, 5 ng/mL) for 6 days. Cell proliferation of HSCs was monitored by a Cell-IQ system (Chipman Technologies, Tampere, Finland), which is a live cell imaging and analysis platform. Cell-IQ automatically recognizes cell type and records corresponding cell number every 15 minutes. Four high-power fields (hpf, 400×magnification) per well were chosen for consecutive cell monitoring and proliferation analysis. The cell proliferation rate was calculated using the following formula: (mean cell number in each hpf after incubation with stimulants)/(mean cell number in each hpf before incubation)×100%−1.

### Flow Cytometry

All antibodies used in flow cytometry were purchased from eBioscience (San Diego, CA, USA), with the exception of PE-conjugated anti-human TGF-β1, 2 and 3 (R&D Systems). For the staining of intracellular cytokines including IL-17, TGF-β and IL-10, cells were stimulated with phorbol-12-myristate-13-acetate (PMA, 25 ng/mL, Enzo, New York, NY, USA) and ionomycin (1 µg/mL, Enzo) in 1 mL RPMI 1640 medium supplemented with 10% FCS at 37°C for 6 hours. Brefeldin A (1 µg/mL, Enzo) was added 1 hour prior to cell harvesting. After labeling with surface antibodies, cells were permeabilized with a fix/perm solution (eBioscience) and stained with the appropriate intracellular antibodies according to the manufacturer’s instructions. Isotype-matched control antibodies were used to determine the level of background staining and help set a gate. Stained cells were analyzed by FACSCalibur (Becton Dickinson) and FlowJo software 7.6.1 (Tristar, El Segundo, CA, USA).

### Western Blot Analysis

HSCs were collected as adherent cells after the co-cultures with CD4^+^, CD4^+^CD25^+^ or CD4^+^CD25^−^ cells from age-and-sex matched HC and CHB subjects or the incubations with various concentrations of human recombinant IL-17 for 3 days. Cell lysis, protein extraction and western blot analysis were performed as described elsewhere [27]. Detection of α-SMA proteins was performed with a rabbit anti-human α-SMA polyclonal antibody (1∶400; Ab5694). Protein loading was normalized using a HRP-conjugated anti-GAPDH antibody (1∶10,000; KC5G5, CHN). The ratio of α-SMA to GAPDH was calculated as the relative quantification.

### In Vivo Administration of Neutralizing Antibodies

To deplete CD25^+^ cells *in vivo*, one day before and after the administration of PBS (controls) or ConA, BALB/c mice were intraperitoneally (i.p.) injected with anti-mouse-CD25 monoclonal antibody (mAb, clone PC61.5.3, rat IgG1, American Type Culture Collection, 0.5 mg/mouse) [28], [29]. To deplete IL-17^+^ cells *in vivo*, anti-IL-17 mAb (Clone 50104, rat IgG2a, R&D Systems, 100 µg/mouse) was also i.p. injected twice a week for up to 4 weeks [30], [31]. Control mice were i.p. injected with control rat IgG (R&D Systems). Cell depletion was confirmed weekly by flow cytometry one day after the second administration of mAb, which always resulted in >90% cell depletion.

### Quantitative Reverse Transcriptase-polymerase Chain Reaction (qRT-PCR)

Mice were scarificed at week four after ConA administration. Their livers were dissected, immediately frozen in liquid nitrogen, and stored at −80°C. Total RNA was extracted using TRIzol (Invitrogen, Carlsbad, CA, USA). Following the manufacturer’s instructions, reverse transcription was performed using a PrimeScript RT reagent kit with gDNA Eraser (Takara, Beijing, China) and quantitative real-time PCR conducted with a SYBR RT-PCR Kit (Takara) using the following conditions: 30 seconds at 95°C, followed by a total of 40 two-temperature cycles (5 seconds at 95°C and 30 seconds at 60°C). Each assay was performed in triplicate. For analysis, the expression of target genes was normalized by the housekeeping gene GAPDH. Based on the ΔΔCt method, relative amounts of mRNA were expressed as 2^−ΔΔCt^. The primer sequences used were as follows: GAPDH sense 5′-TGTGTCCGTCGTGGATCTGA-3′; GAPDH antisense 5′-TTGCTGT TGAAGTCGCAGGAG-3′; collagen type I alpha 1 (COL1a1) sense 5′-TGCTGGCCCCAAG GGTCCTT-3′; COL1a1 antisense 5′-GGCTGCCAGGACTGCCA GTG-3′; collagen type III alpha 1(COL3a1) sense 5′-CTTAGAAGCTGATGGGATC-3′; COL3a1 antisense 5′-TTGCCTTGCGTG TTTGT-3′; TGF-β1 sense 5′-GTGTGGAGCAACATGTGGAAC TCTA-3′; TGF-β1 antisense 5′-TTGGTTCAGCCACTGCCGTA-3′; α-SMA sense 5′-AAGAG CATCCGACACTGCTGAC-3′; α-SMA antisense 5′-AGCACAGCCTGAATAGCCACATAC-3′; PDGF-BB sense 5′-GAGATT GTGCGAAAGAAGCC-3′; PDGF-BB antisense 5′-CTTCTAGTCACAGGCCGAGG-3′.

### Statistical Analysis

Results are presented as mean ± standard error of the mean (SEM), in triplicate. Statistical analyses were performed using the GraphPad Software Version 5.01 (CA, USA). Student’s *t-*test, one-way ANOVA, χ^2^ test and Pearson’s rank correlation were performed as appropriate, and *p* values of less than 0.05 (two-tailed) were considered statistically significant.

## Results

### Phenotypes of Treg and Th17 Cells in the Periphery of CHB Patients

**Figure 1:**
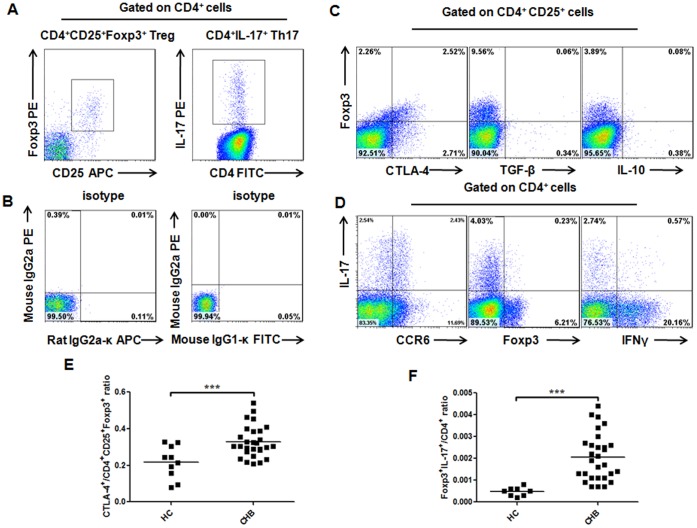
Phenotypes of Treg and Th17 cells in the periphery of CHB patients. (A, B) Representative dot plots of peripheral Treg and Th17 cells with matched-isotype controls. (C) Representative co-expressions of CTLA-4, IL-10 and TGF-β in Treg cells of CHB patients. (D) Representative co-expressions of CCR6, IFN-γ and Foxp3 in Th17 cells of CHB patients. (E) The frequencies of CD4^+^CD25^+^Foxp3^+^CTLA-4^+^ cells are significantly higher in CHB patients (*n* = 28) than in HC subjects (*n* = 10). (F) The frequencies of CD4^+^Foxp3^+^IL-17^+^ cells are significantly higher in CHB patients (*n* = 29) than in HC subjects (*n* = 9). Data in subfigures D–E are presented as each value and the mean value. ***, *p*<0.001.

We characterized CD4^+^CD25^+^Foxp3^+^ and CD4^+^IL-17^+^ cells respectively as Treg and Th17 cells, (Figure1A), and determined their ratios in total CD4^+^ cells as the frequencies. The isotype controls were also shown (Figure 1B). We selectively determined the co-expression of membrane-bound inhibitory molecule CTLA-4, and inhibitory cytokines IL-10 and TGF-β in Treg cells from CHB patients (Figure 1C). Treg cells from CHB patients expressed significantly more CTLA-4 than did those from HC subjects (Figure 1E). However, Treg cells whether from CHB patients or HC subjects barely secreted any IL-10 or TGF-β. We also determined the co-expression of chemokine receptor-6 (CCR6, home receptor), IFN-γ and Foxp3 in Th17 cells. Results were that in CHB patients, a plurality of Th17 cells (32%–72%, n = 20) co-expressed CCR6, whereas much less of them co-expressed IFN-γ or Foxp3 (Figure 1D). In addition, we found that the frequency of CD4^+^IL-17^+^Foxp3^+^ cells significantly increased in CHB patients (Figure 1F).

### Treg and Th17 Cells Involved in Fibrosis Progression of HBV-LF

**Figure 2:**
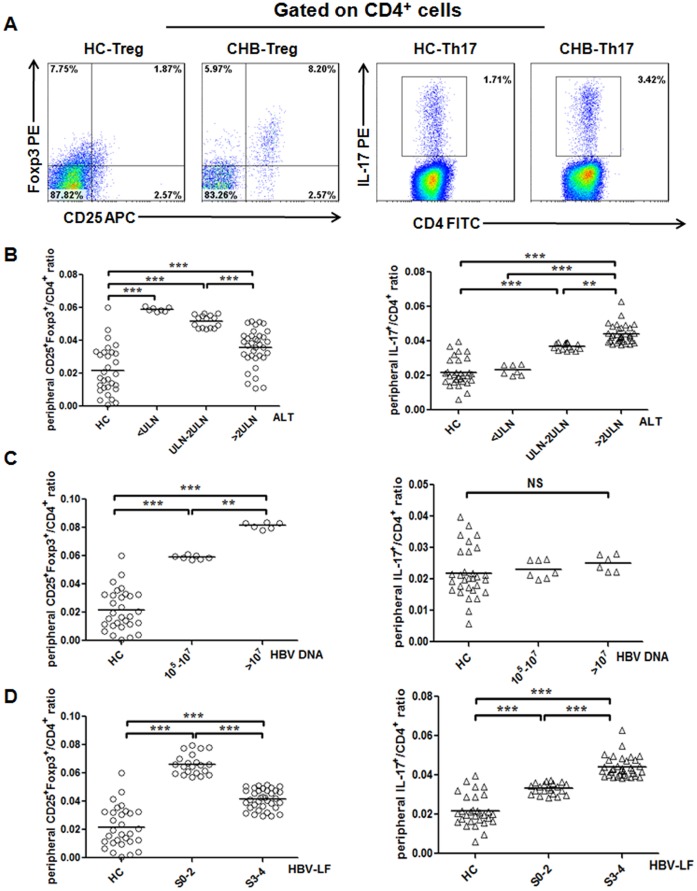
Treg and Th17 cells involved in fibrosis progression of HBV-LF. (A) Representative dot plots of Treg (right panel) and Th17 (left panel) cells in age-and-sex matched HC and CHB subjects. (B) Subgroup analyses were performed in CHB patients with similar HBV loads (10^5^–10^7^ copies/mL) but with differing ALT levels (*n* = 57). With elevated ALT levels, Treg frequency decreased significantly whereas Th17 increased significantly. (C) Subgroup analyses were performed in CHB patients with normal ALT but differing virus loads (*n* = 13). With elevated virus loads, Treg frequency increased significantly whereas Th17 barely changed. (D) Subgroup analyses were performed in CHB patients with similar inflammation grades (G2–3) but with differing stages of fibrosis (*n* = 56). With elevated fibrosis stages, Treg frequency decreased significantly whereas Th17 increased significantly. Data in subfigures B–D are presented as each value and the mean value. **, *p*<0.01; ***, *p*<0.001; NS, not significant.

We determined the frequencies of Treg and Th17 cells in the periphery of 30 HC subjects and 77 CHB patients by flow cytometry. Compared to HC subjects, both Treg and Th17 cell frequencies significantly increased in CHB patients (Figure 2A). Next, we analyzed Treg and Th17 cell frequencies in differently-classified CHB subgroups, and found in CHB patients: with increased ALT levels, Treg frequency down-regulated whereas Th17 frequency up-regulated, both significantly (Figure 2B); with increased HBV loads, we observed a significant increase of Treg frequency but no change of Th17 frequency (Figure 2C); and with increased S scores, we detected a decrease of Treg frequency and an increase of Th17 frequency both in dramatic amplitudes (Figure 2D).

### Peripheral Treg/Th17 Ratios Indicated Liver Injury and Fibrosis Progression of HBV-LF

**Figure 3:**
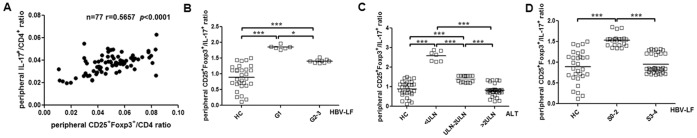
Peripheral Treg/Th17 ratios indicated liver injury and fibrosis progression of HBV-LF. A) There is a significant correlation between peripheral Treg and Th17 frequencies in CHB patients. (n, case number; r, correlation coefficient; *p* value is shown). (B–D) Compared to HC subjects, peripheral Treg/Th17 ratio dramatically increased in CHB patients, however it dramatically decreased with enhanced inflammation grades (B) or elevated ALT levels (C) or advanced fibrosis stages (D). Data in subfigures B–D are presented as each value and the mean value. *, *p*<0.05; ***, *p*<0.001.

Regulatory Treg and Th17 cells have been reportedly closely-linked [4], [7], [8]. Here we first performed the Pearson’s rank correlation between peripheral Treg and Th17 frequencies of CHB patients (*n* = 77) and found these two cell lineages were significantly correlated (r = 0.5657, *p<*0.0001) (Figure 3A), suggesting the existence of Treg/Th17 balance in CHB patients. We then calculated the Treg/Th17 ratios using the frequencies and found that the ratios of CHB patients were dramatically higher than those of HC subjects. However, in CHB subgroups, the ratios significantly decreased whether with enhanced G scores (Figure 3B) or increased ALT levels (Figure 3C) or with advanced S scores (Figure 3D). The ratios did not change dramatically with elevated virus loads (data not shown).

### Treg Cells Regulated Th17 Response in HBV-LF

**Figure 4:**
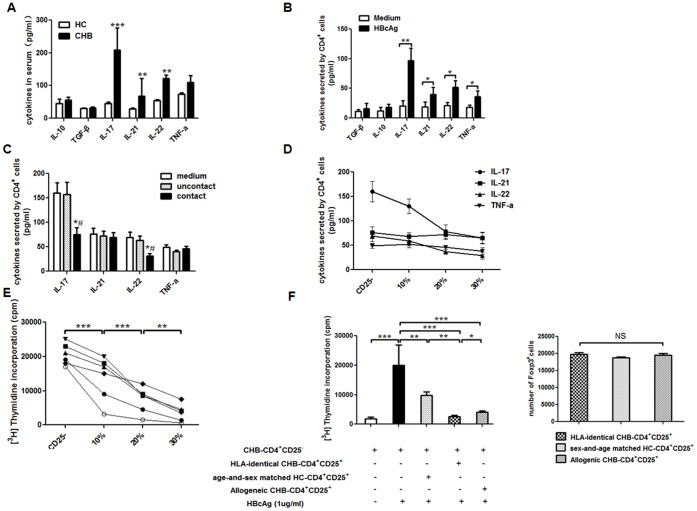
Treg cells regulated Th17 response in HBV-LF . (A) Compared to HC subjects (*n* = 30), Th17-associated cytokines such as IL-17, IL-21 and IL-22 significantly increased in serum of CHB patients (*n* = 77), however Treg-associated inhibitory cytokines IL-10 and TGF-β are similar in HC subjects and CHB patients. (B) After 1 µg/mL HBcAg stimulation for 5 days, CD4^+^ cells from CHB patients (*n* = 6) showed a significantly enhanced ability to secrete Th17-associated cytokines, including IL-17, IL-21, IL-22 and TNF-α, than do those from age-and-sex matched HC subjects (*n* = 6). (C) CD4^+^CD25^+^ cells from CHB patients (*n* = 6) were co-cultured with HLA-identical CD4^+^CD25^−^ cells in both contact and non-contact manners for 5 days. CD4^+^CD25^+^ cells significantly inhibited IL-17 and IL-22 production by CD4^+^CD25^−^ cells only in cell-contact manner. *, *p*<0.05 when compared to medium only group; ^#^, *p*<0.05 when compared to uncontact co-culture group. (D) CD4^+^CD25^+^ cells from CHB patients (*n* = 6) significantly inhibited IL-17 and IL-22 production by CD4^+^CD25^−^ cells in a dose-dependent manner (*p*<0.05). No dramatic changes of IL-21 or TNF-α were observed after co-cultures with CD4^+^CD25^+^ cells. Each line represents a cytokine (*p*>0.05). (E) CD4^+^CD25^+^ cells from CHB patients (*n* = 6) inhibited proliferation of HLA-identical CD4^+^CD25^−^ cells in a dose-dependent manner. Each line represents a CHB patient. (F) On the left panel, HLA-identical CD4^+^CD25^+^ cells showed the greatest power to inhibit proliferation of CD4^+^CD25^−^ cells compared to allogenic CD4^+^CD25^+^ cells whether from other CHB patients or age-and-sex matched HC subjects (*n* = 6). On the right panel, the number of Foxp3^+^ cells among different groups of CD4^+^CD25^+^ cells used to co-culture with CD4^+^CD25^−^ cells was clarified. Data in subfigures A, B, C, and E are expressed as the mean ± SEM. *, *p*<0.05; **, *p*<0.01; ***, *p*<0.001.

We determined lineage-associated cytokines of Treg and Th17 cells in the serum of CHB patients and HC subjects, and found the concentrations of IL-17, IL-21 and IL-22 significantly increased in CHB group whereas those of IL-10 and TGF-β were similar in both groups (Figure 4A). In addition, in response to HBcAg, CD4^+^ cells from CHB patients showed a significantly enhanced ability to secret Th17-associated cytokines, including IL-17, IL-21, IL-22 and TNF-α (Figure 4B). Although both Treg and Th17 responses involved in CHB pathogenesis, the above results suggested that Treg cells might be independent of cytokines whereas Th17 cells mainly depended on cytokines. Because Foxp3 staining requires cell membrane permeablization and impairs cell function, CD4^+^CD25^+^ cells are used to represent Treg response to perform following *in vitro* experiments as described elsewhere [4]–[6]. To investigate the influence of Treg cells on Th17 response during HBV-LF, with the adding of 1 µg/mL HBcAg during co-cultures, CD4^+^CD25^+^ cells (2.5×10^4^ cells/well) from CHB patients (*n* = 6) were co-cultured with HLA-identical CD4^+^CD25^−^ cells (1×10^5^/well) in both contact and non-contact manners for 5 days at a cell ratio of 1∶4, and Th17 associated cytokines in the supernatant were determined by ELISA. We found the CD4^+^CD25^+^ cells from CHB patients could significantly down-regulate the concentrations of IL-17 and IL-22 in the co-culture systems, which were probably produced by Th17 cells existing in CD4^+^CD25^−^ cells, and this effect was only observed when the co-cultures were performed in contact manner (Figure 4C). Therefore, we performed subsequent co-cultures only in contact manner. Next, also with 1 µg/ml HBcAg, the inhibition of CD4^+^CD25^+^ cells on Th17 response was performed by directly adding 0, 10% (11,000/well), 20% (25,000/well), or 30% (43,000/well) CD4^+^CD25^+^ cells from CHB patients (n = 6) to HLA-identical CD4^+^CD25^−^ cells (1×10^5^/well) and the contact co-cultures were performed for 6 days. We found that CD4^+^CD25^+^ cells from CHB patients dramatically inhibited IL-17 and IL-22 production by CD4^+^CD25^−^ cells (Figure 4D) and the proliferation of CD4^+^CD25^−^ cells both in dose dependent manners (Figure 4E). We also performed some co-cultures of CD4^+^CD25^−^ cells (1×10^5^/well) from CHB patients together with HLA-identical CD4^+^CD25^+^ cells or allogenic CD4^+^CD25^+^ cells (25,000/well) from other CHB patients or age-and-sex matched HC subjects (*n* = 6). We also determined the number of Foxp3^+^ cells among different groups of CD4^+^CD25^+^ cells by flowcytometry, and found that containing almost the same number of Foxp3^+^ cells, HLA-identical CD4^+^CD25^+^ cells showed the most enhanced ability to inhibit the proliferation of CHB-CD4^+^CD25^−^ cells in response to HBcAg (Figure 4F).

### CD4^+^ Cells During HBV-LF Promoted Activation and Proliferation of Primary HSCs

**Figure 5:**
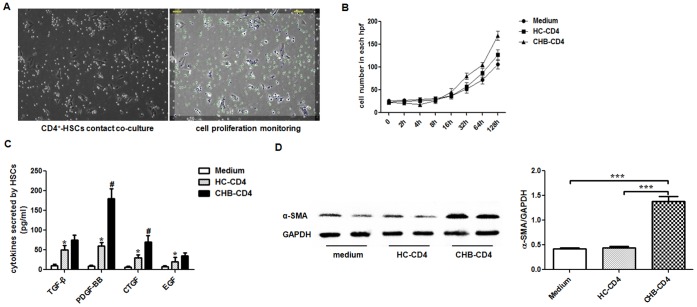
CD4^+^ cells during HBV-LF promoted activation and proliferation of primary HSCs. (A) Co-cultures between HSCs and CD4^+^ cells were monitored by a Cell-IQ® system: HSCs were marked as blue dots and CD4^+^ cells as green ones. (B) Growth curves of HSCs cultured alone or with CD4^+^ cells from age-and-sex matched HC and CHB subjects for 128 consecutive hours (*n* = 6). (C) The concentrations of TGF-β, PDGF-BB, CTGF and EGF in the supernatant at day 6 after co-cultures between HSCs and CD4^+^ cells from age-and-sex matched HC and CHB subjects (*n* = 6). (D) The α-SMA proteins in HSCs that were contactly co-cultured with CD4^+^ cells from age-and-sex matched HC and CHB subjects (*n* = 6) were analyzed by western blot and the relative quantification was also shown. Data in subfigures C and D are expressed as the mean ± SEM. *, *p*<0.05 compared to medium only group; ^#^, *p*<0.05 compared to the HC-CD4 group; ***, *p*<0.001.

Over the decade, activation of HSCs has always been the cornerstone of liver fibrosis [15]. Activated HSCs usually proliferate more dramatically than their quiescent forms. Here, to investigate the influence of CD4^+^ cells on HSCs during HBV-LF, we first performed contact co-cultures of primary HSCs (1×10^5^/well) in intermediate state respectively with CD4^+^ cells (1×10^4^/well) from age-and-sex matched CHB and HC subjects (*n* = 6) for 6 days. We consecutively monitored cell proliferation of HSCs with the Cell-IQ (Figure 5A) and assessed HSC-sourced growth factors (TGF-β, PDGF-BB, CTGF and EGF) in the supernatant at the end of the co-cultures via ELISA. Data indicated that, compared to HC subjects, CD4^+^ cells from CHB patients dramatically promoted cell proliferation (Figure 5B) and growth factors production of HSCs (Figure 5C). Also, by western blot, we determined α-SMA proteins in adherent cells, as differently-cultured HSCs, at the end of co-cultures. We found CD4^+^ cells from CHB patients promoted, whereas those from HC subjects barely regulated α-SMA proteins in HSCs (Figure 5D).

### Treg and Th17 Responses During HBV-LF Differently Regulated Primary HSCs in Vitro

**Figure 6:**
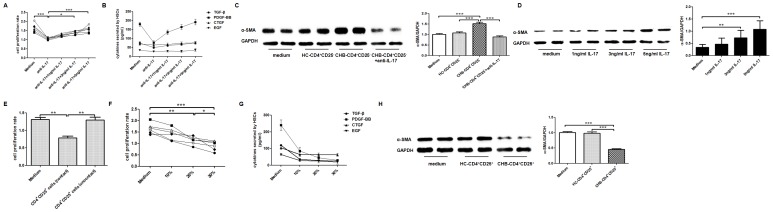
Treg and Th17 responses during HBV-LF differently regulated primary HSCs *in vitro.* (A) IL-17 blocking dramatically inhibited the proliferation of HSCs, and re-addition of recombinant IL-17 enhanced HSCs proliferation in a dose-dependent manner (*n* = 6). Each line represents a CHB patient. (B) Recombinant IL-17 promoted PDGF-BB production by HSCs (*n* = 6). Each line represents a growth factor. (C) The α-SMA proteins in HSCs after co-cultures with CD4^+^CD25^−^ cells from age-and-sex matched HC and CHB subjects at day six determined by western blot, with or without IL-17 blocking, are presented and the relative quantification is shown (*n* = 6). (D) The α-SMA proteins in HSCs after IL-17 incubation at different concentrations for six days determined by western blot are presented in the right panel and the relative quantification is in the left panel (*n* = 3). (E) CD4^+^CD25^+^ cells inhibit the proliferation of HSCs in cell contact manner (*n* = 3). Data are expressed as the mean ± SEM. (F) CD4^+^CD25^+^ cells from CHB patients inhibited cell proliferation of HSCs in a dose-dependent manner (*n* = 6). Each line represents a CHB patient. (G) CD4^+^CD25^+^ cells from CHB patients inhibited PDGF-BB and TGF-β production of HSCs in dose-dependent manners (*n* = 6). Each line represents a growth factor. (H) The α-SMA proteins in HSCs after co-cultures with CD4^+^CD25^+^ cells from age-and-sex matched HC and CHB subjects at day six are presented and the relative quantification is shown (*n* = 6). Data in subfigures C, D, E&H are expressed as the mean ± SEM *, *p*<0.05; **, *p*<0.01; ***, *p*<0.001.

Since no reliable surface markers have been identified for Th17 cells yet, we used recombinant IL-17 (PeproTech) cytokine to partially represent Th17 response in our study. To detect influence of Th17 response on HSCs during HBV-LF, we cultured HSCs (1×10^5^/well) in contact manner with CD4^+^CD25^−^ cells (1×10^4^/well) from CHB patients (*n* = 6) with or without adding anti-IL-17 antibody (10 µg/mL) and recombinant IL-17 at various concentrations (1, 3, 5 ng/mL) for 6 days. Results indicated that Th17 response of CHB patients enhanced cell proliferation and PDGF-BB production of HSCs both in dose-dependent manners (Figure 6A&B). We then co-cultured HSCs (1×10^5^/well) respectively with CD4^+^CD25^−^ cells (1×10^4^/well) from age-and-sex matched HC and CHB subjects (*n* = 6), also with the addition of anti-IL-17 (10 µg/mL) to CHB-CD4^+^CD25^−^ cells, for 6 days, and determined α-SMA proteins in adherent HSCs by western blot. As we expected, compared to HC-CD4^+^CD25^−^ cells, CHB-CD4^+^CD25^−^ cells could dramatically up-regulated α-SMA proteins in HSCs, and anti-IL-17 antibody could dramatically weaken this up-regulation effect (Figure 6C). The exact effect of IL-17 cytokine on activation of HSCs was further proved by the incubation of HSCs with recombinant IL-17 (1, 3, 5 ng/mL) for 6 days. And we observed IL-17 significantly up-regulated α-SMA proteins of HSCs in a dose-dependent manner (Figure 6D). To detect influence of Treg response on HSCs, we firstly cultured CD4^+^CD25^+^ cells (1×10^4^/well) from CHB patients with HSCs in both contact and non-contact manners for 6 days. Data showed that CD4^+^CD25^+^ cells could only inhibit proliferation of HSCs in contact co-cultures (Figure 6E). Besides, we determine the inhibition of allogenic CD4^+^CD25^+^ cells from CHB patients on HSCs by directly adding 10% (11,000/well), 20% (25,000/well), or 30% (43,000/well) CD4^+^CD25^+^ cells to HSCs (1×10^5^/well) and co-culturing them in contact manner for 6 days., and demonstrated CD4^+^CD25^+^ cells dramatically inhibited cell proliferation and PDGF-BB and TGF-β production of HSCs both in dose-dependent manners (Figure 6F&G). Moreover, we respectively co-cultured HSCs (1×10^5^/well) with CD4^+^CD25^+^ cells (1×10^4^/well) from age-and-sex matched HC and CHB subjects (n = 6) for 6 days and detected α-SMA proteins in HSCs at the end of co-cultures, and found that CHB-CD4^+^CD25^+^ cells significantly inhibited the expression of α-SMA proteins by HSCs in comparison with HC-CD4^+^CD25^+^ cells (Figure 6H).

### Treg/Th17 Balance Regulates Liver Fibrosis Progression in Mouse Models

**Figure 7:**
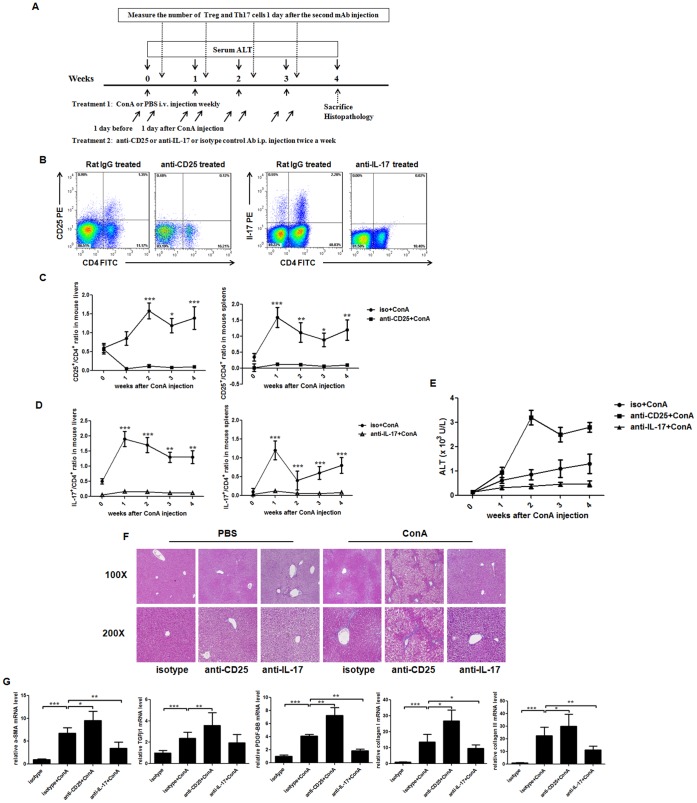
Treg/Th17 balance regulated liver fibrosis progression in mouse models . (A) Experimental protocol: Mice were i.v. injected with ConA weekly for up to four weeks to establish liver fibrosis models. Depletion of CD25^+^ or IL-17^+^ cells during fibrosis progression was performed with i.p. injection of either anti-CD25 or anti-IL-17 mAb one day before and after ConA injection twice a week. (B) Representative dot plots of CD4^+^CD25^+^ or CD4^+^IL-17^+^ cells in mouse livers are shown at week 4 after the administration of anti-CD25 or anti-IL-17 or isotype control mAb. (C) The frequencies of CD4^+^CD25^+^ cells in mouse livers and spleens with administration of anti-CD25 or isotype control mAb were determined at week 0, 1, 2, 3 and 4 after ConA injection. ***, *p*<0.001 compared to isotype at week 0. (D) The frequencies of CD4^+^IL-17^+^ cells in mouse livers and spleens with administration of anti-IL-17 or isotype control mAb were determined at week 0, 1, 2, 3 and 4 after ConA injection. **, *p*<0.01; ***, *p*<0.001 compared to isotype at week 0. (E) Serum ALT levels at week 0, 1, 2, 3 and 4 after ConA injection. (F) Representative Masson staining of livers in ConA or PBS treated mice is shown at week 4 after the administration of anti-CD25 or anti-IL-17 or isotype Ab (× 100 and × 200 magnification). *n* = 6–8 per group. (G) The mRNA levels of α-SMA, PDGF-BB, TGF-β, collagen type I and III in mouse livers at week 4 after ConA injection are shown in four groups: isotype, isotype + ConA, anti-CD25+ ConA and anti-IL-17+ ConA groups. Data are expressed as the mean ± SEM; *, *p*<0.05; **, *p*<0.01; ***, *p*<0.001.

The above data from CHB patients demonstrated that both Treg and Th17 responses were significantly correlated with liver injury and fibrosis degree of HBV-LF, and differently regulated cell proliferation and activation of HSCs, suggesting the Treg/Th17 balance as an uncharacterized mechanism to regulate HBV-LF progression. To gain *in vivo* evidence of the Treg/Th17 balance during fibrosis progression, in this part we firstly established ConA-induced mouse liver fibrosis models (Figure7A) and found the frequencies of CD25^+^ and IL-17^+^ cells significantly increased in livers and spleens after ConA administrations (Figure 7B–D), which was similar with the increase of Treg and Th17 frequencies in CHB patients. Then we depleted CD25^+^ or IL-17^+^ cells by administering anti-CD25 mAb or anti-IL-17 mAb twice a week (one day before and after ConA administration) in mouse models (Figure 7A–D). Cell depletion was confirmed once a week by the frequencies of CD25^+^ or IL-17^+^ cell in livers and spleens using flow cytometry one day after the second mAb administration, and the frequencies of both cell types in mouse livers and spleens showed dramatic decreases after mAb injections (Figure 7B–D). We also measured serum ALT levels and found that ConA injection enhanced ALT activity. The administration of anti-CD25 mAb further increased whereas that of anti-IL-17 mAb decreased ALT levels (Figure 7E). We moreover performed histopathological analyses with Masson staining at week 4 after ConA injections and found that, compared to the isotype-and-ConA treated mice, the anti-CD25-and-ConA treated mice had intensified liver fibrosis whereas anti-IL-17-and-ConA treated mice showed alleviated liver fibrosis (Figure 7F). We further confirmed these results by detecting the pro-fibrotic genes such as α-SMA, PDGF-BB, TGF-β1, collagen type I and III, and observed that, both significantly, anti-CD25-and-ConA treated mice up-regulated whereas anti-IL-17-and-ConA treated mice down-regulated the above genes in liver tissues (Figure 7G).

## Discussion

Although increasing evidence suggests that inappropriate inflammation drives progression of fibrosis, cellular interactions between inflammation and fibrosis in HBV-LF are poorly understood [20]. Recently, much attention has focused on imbalance between Treg cells and other effector T cells as a mechanism of inappropriate inflammation [7]–[8], [10]–[14]. Both Treg and Th17 responses have been described in CHB patients [4]–[6], [9]–[10], [14], however the significance of the Treg/Th17 balance during fibrosis progression of HBV-LF has not been determined.

First, we performed several phenotype studies on peripheral Treg and Th17 cells in CHB patients and found that Treg cells from CHB patients expressed more CTLA4 but produced very limited IL-10 and TGF-β1. Although we did not determine GITR expression by Treg cells in our study, our data had already demonstrated the inhibitory ability of these cells was not dependent on cytokines, for Treg-associated cytokines (IL-10 and TGF-β) were barely detected whether in CD4^+^CD25^+^Foxp3^+^ cells intracellularly after PMA/ionomycin stimulation, or in serum samples, or in the supernatant of cultured-CD4^+^ cells after HBcAg incubation, and moreover, CD4^+^CD25^+^ cells from CHB patients could only inhibit IL-17 and IL-22 production of CD4^+^CD25^−^ cells in contact co-cultures. In addition, the considerable co-expression of CCR6 on peripheral Th17 cells in CHB patients suggested the involvement of CCR6-CCL20 (CCR6 ligand) pathway in CHB pathogenesis. Moreover, we also documented a significantly higher frequency of peripheral CD4^+^Foxp3^+^IL-17^+^ cells in CHB patients. Because CD4^+^Foxp3^+^IL-17^+^ cells may exist as a stable population with shared functions of both Treg and Th17 cells, or as a transient population that may eventually generate either Treg or Th17 cells, which had also been reported by other researchers [4], [7], [9], these cells present a potential mechanism to regulate the Treg/Th17 balance during HBV-LF. However, future researches need to fully elucidate all these mechanisms.

In the present study, we observed that both Treg and Th17 frequencies in the periphery of CHB patients were much higher than those of HC subjects, which coincides with other researches [4]–[6], [9]–[10], [14] and further supports the involvement of Treg and Th17 cells in CHB pathogenesis. To further investigate effects of Treg and Th17 responses in HBV-LF, we rigorously classified CHB patients with certain clinical parameters into subgroups according to their ALT levels, HBV loads and fibrosis degrees. In the periphery of CHB patients, we found Treg frequency positively correlated with HBV load whereas negatively correlated with liver injury and fibrosis degree. In contrast, we observed a significantly positive correlation of Th17 frequency with liver injury and fibrosis degree of CHB patients, but no significant correlation between Th17 frequency and HBV load was detected.

In CHB patients, although Treg cells could favor HBV replication, they might be a protective factor of HBV-LF for: 1) they could down-regulate inflammation to alleviate liver injury and 2) they could inhibit the activation and proliferation of HSCs, the cornerstone of liver fibrosis, during in-vitro co-cultures. The exact significance of Treg cells in HBV infection might depend on their presenting time: if they are aroused by HBV antigens in early stage of virus infection, they might protect HBV thus being “harmful”, however if they are induced by hosts in late phase of virus infection to limit excessive inflammation, they might prevent liver injury thus being “helpful”. The existence of various subsets of Treg cells with different functions could explain the double role they play, which have been reported in several researches [2], [6]–[8]. Since we only included CHB patients in this study, we could dissect the presenting time of these Treg cells. Future studies should undertake lineage-based studies of Treg cells at different time points of HBV-LF.

On the effects of Th17 cells on CHB pathogenesis, we supposed they might be a progressive factor of HBV-LF since we observed a significantly correlation of Th17 frequency with liver injury and fibrosis degree, no significant correlation between Th17 frequency and HBV load, and moreover, IL-17 cytokines mainly produced by Th17 cells could dramatically promote the activation and proliferation of HSCs, the cornerstone of liver fibrosis. However, HBV-specific Th17 cells had also been reported to play a role in HBV clearance [10]. The data inconsistency might result from different inclusion criteria of CHB patients in different studies, thus the significance of Th17 cells in HBV-LF need to be determined in the future.

In CHB patients, we firstly found a significant correlation between Treg and Th17 frequencies in CHB patients, indicating the close link of Treg and Th17 cells in CHB pathogenesis. We therefore calculated the Treg/Th17 ratios in CHB patients, an indicator of the balance between Treg and Th17 cells [10], [12]–[14], and presented it as an indicator of inflammation degree in this process for 1) Treg and Th17 cells are both involved in CHB pathogenesis and respectively known to down-regulate and up-regulate the inflammation; and 2) the Treg/Th17 ratio are negatively correlated with the inflammation degree of CHB patients. This ratio is significantly higher in CHB patients compared to HC subjects, which means anti-inflammation overrides pro-inflammation in CHB pathogenesis, suggesting the immune-tolerant state is required to sustain chronic HBV infection. However, when liver fibrosis progresses in CHB patients, this ratio dramatically decreased, indicating the persistent and progressive inflammation is essential for fibrosis progression. All these indicated the Treg/Th17 balance as an uncharacterized mechanism to regulate HBV-LF progression.

We also analyzed lineage-associated cytokines of Treg and Th17 cells [2], [7]–[9] in serum of CHB patients, and found that Th17-associated pro-inflammatory cytokines (IL-17, IL-22, IL-21 and TNF-α) were enriched whereas Treg-associated anti-inflammatory cytokines (IL-10 and TGF-β) were barely detected. Moreover, in response to HBcAg, CD4^+^CD25^−^ cells from CHB patients showed an enhanced ability to produce IL-17, IL-22, IL-21 or TNF-α rather than IL-10 or TGF-β, whereas CD4^+^CD25^+^ cells barely produced these cytokines (data not shown), so CD4^+^CD25^−^ cells are supposed to contain almost all HBV-specific Th17 cells. These results also indicated the cytokine-independence of Treg cells and the cytokine-dependence of Th17 cells in CHB pathogenesis. Moreover, CD4^+^CD25^+^ cells could dramatically inhibit cytokine production and proliferation of HLA-identical CD4^+^CD25^−^ cells in a dose-dependent manner but only during contact co-cultures, indicating the requirement of cell contacts in Treg response. This part of research suggested the existence of Treg/Th17 balance from the functional connections that Treg cells could down-regulate Th17 response in vitro. However, further *in vivo* evidence should be obtained. Interestingly, with almost the same number of Foxp3^+^ cells, HLA-identical CD4^+^CD25^+^ cells rather than allogenic CD4^+^CD25^+^ cells aroused the strongest inhibition on proliferation of CD4^+^CD25^−^ cells in response to HBcAg, indicating the functional diversity of these CD4^+^CD25^+^ cells for the following possible mechanisms: 1) Treg response in HC subjects was not shaped by HBV-related antigens thus been different from that in CHB patients; 2) HBV-related antigens induced exclusive Treg response in each CHB individual for the existence of various modes of host-virus interactions. So, the ideal immune therapy for CHB patients should be based on individualization.

Over the past decade, the activation of HSCs into myofibroblasts has always been critical to liver fibrogenesis [15]. We therefore designed a series of co-cultures of different CD4^+^ cell lineages with HSCs to explore exact effects of different CD4^+^ cell responses on HSCs activation and proliferation. Total CD4^+^ cells include many functional lineages, including Th1, Th2, Treg, Th17 and even Th0 cells. Thus, data from CD4^+^ cells reflect the effects of complicated interactions among various lineages. From an overall perspective, we demonstrated that total CD4^+^ cells from CHB patients dramatically promoted cell proliferation, α-SMA expression and growth factors production (TGF-β, PDGF-BB, CTGF and EGF) of HSCs, thus promoting liver fibrosis progression. Then CD4^+^ cells were dissected into CD4^+^CD25^+^ and CD4^+^CD25^−^ cells to perform respective co-cultures with HSCs, and we observed absolutely opposite effects of these two CD4^+^ cell lineages on the activation of HSCs, for CD4^+^CD25^+^ cells inhibited whereas CD4^+^CD25^−^ cells promoted α-SMA expression. In addition, we found that during contact cultures, CD4^+^CD25^+^ cells could inhibit cell proliferation and growth factors production of HSCs in a dose-dependent manner. Moreover, the effect of IL-17 response induced by Th17 cells on HSCs was determined using CD4^+^CD25^−^ cells in combination of anti-IL-17 antibody and recombinant IL-17 cytokine, and we found that IL-17 neutralization down-regulated whereas IL-17 addition up-regulated both cell proliferation and growth factors production of HSCs in a significantly dose-dependent manner. These data again suggested the Treg/Th17 imbalance as an uncharacterized mechanism to regulate fibrosis progression, for Treg and Th17 responses directly-regulate the pro-fibrotic features of HSCs in an adverse manner, and also, Treg cells indirectly influence HSCs via regulation of Th17 response.

To obtain *in vivo* evidence about effect of Treg/Th17 balance in liver fibrosis, we established ConA-induced mouse liver fibrosis models, characterized by the infiltration of abundant CD4^+^ cells to induce immune-mediated liver fibrosis. We found that the frequencies of CD4^+^CD25^+^ and CD4^+^IL-17^+^ cells significantly increased in mouse livers and spleens after ConA administration, suggesting the involvement of Treg and Th17 responses in ConA-induced liver fibrosis. This finding provides a premise to investigate effect of the Treg/Th17 balance in fibrosis models via depletion of CD25^+^ or IL-17^+^ cells. We also observed that, in mouse livers, the peak of CD4^+^ IL-17^+^ cell frequency occurred at week 1, which was earlier than appearing time of the peak of ALT level and CD4^+^CD25^+^ cell frequency (both at week 2). Based on what has already been established about CD4^+^CD25^+^ and CD4^+^ IL-17^+^ cells in mice [28]–[31], this phenomenon can be easily explained as follows: ConA injection induces an increase in CD4^+^ IL-17^+^ cells, which cause severe liver injury, which in turn induces CD4^+^CD25^+^ cells to constrain the Th17 response or directly alleviate cell injury. From the perspective of liver injury, it is easy to understand the resulting histopathology that the depletion of CD25^+^ cells promoted whereas the depletion of IL-17^+^ cells inhibited fibrosis progression. The protective role of CD4^+^CD25^+^ cells and the progressive role of CD4^+^ IL-17^+^ cells during liver fibrosis were then demonstrated by mRNA levels of the pro-fibrotic genes (α-SMA, TGF-β1, PDGF-BB, collagen I/III) in liver tissues, for the depletion of CD25^+^ cells up-regulated, whereas the depletion of IL-17^+^ cells down-regulated these genes. Thus, we conclude that the Treg/Th17 balance regulates disease progression in ConA-induced fibrosis models.

Collectively, anti-inflammatory Treg cells and pro-inflammatory Th17 cells have antagonistic effects on fibrosis progression in HBV-LF for their influence on liver injury and HSCs function. Here, we do not find a direct association between HBV replication and fibrosis progression, but highlight immune-mediated liver injury during HBV-LF progression. The characterization of the Treg/Th17 balance in HBV-LF by our work paves the way for future researches about inflammation and fibrosis pathways.

## Supporting Information

Figure S1 **Purification of CD4^+^, CD4^+^CD25^−^ and CD4^+^CD25^+^ cells.** The purity of isolated CD4^+^, CD4^+^CD25^+^ and CD4^+^CD25^−^ cells was confirmed >90% by flow cytometry.
(TIF)

Figure S2 **Identification of isolated primary human HSCs.** The purity of isolated HSCs was determined as ranged from 90% to 95% by GFAP staining. At day 7 after *in vitro* culture, HSCs become activated and begin to express a-SMA.
(TIF)
