# Supplementary figures and images for: Significance of the Balance between Regulatory T (Treg) and T Helper 17 (Th17) Cells during Hepatitis B Virus Related Liver Fibrosis
Source: PLoS One. 2012 Jun 20;7(6):e39307. doi: 10.1371/journal.pone.0039307 (PMC3380028; doi:10.1371/journal.pone.0039307)

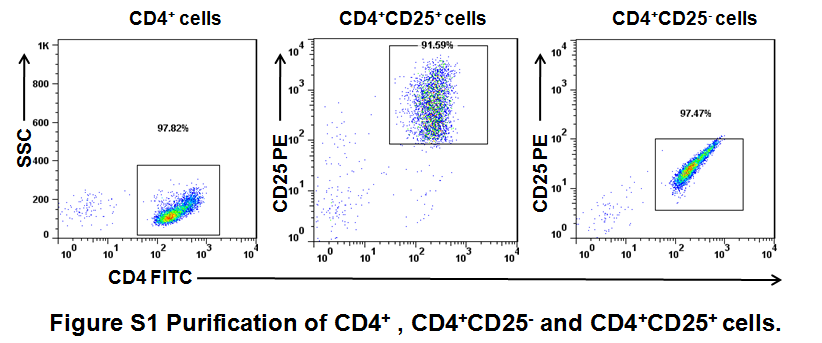

Supplement: Figure S1 — Purification of CD4+, CD4+CD25− and CD4+CD25+ cells. The purity of isolated CD4+, CD4+CD25+ and CD4+CD25− cells was confirmed >90% by flow cytometry. (TIF) [file pone.0039307.s001.tif]

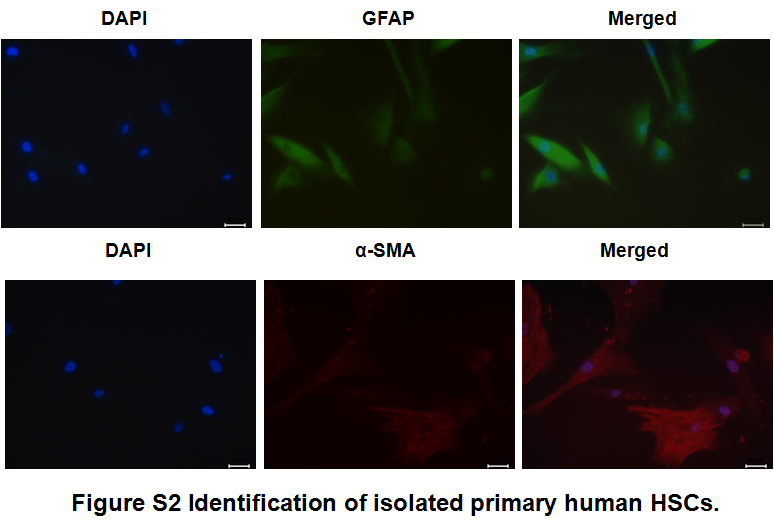

Supplement: Figure S2 — Identification of isolated primary human HSCs. The purity of isolated HSCs was determined as ranged from 90% to 95% by GFAP staining. At day 7 after in vitro culture, HSCs become activated and begin to express a-SMA. (TIF) [file pone.0039307.s002.tif]
